# My tongue hurts

**DOI:** 10.1038/s41415-022-5026-8

**Published:** 2022-10-14

**Authors:** Stephen Porter, Michael Escudier, Stefano Fedele

**Affiliations:** 4141510405001 Institute Director and Professor of Oral Medicine, UCL Eastman Dental Institute, University College London, London, UK; 4141510405002 Professor of Oral Medicine, Faculty of Dentistry, Oral and Craniofacial Sciences, King´s College London, London, UK; 4141510405003 Institute Director and Professor of Oral Medicine, UCL Eastman Dental Institute, University College London, London, UK; UCLH/UCL NIHR Biomedical Research Centre, London, UK

## Abstract

This series of articles comprise of short reviews of clinical problems relevant to oral health care in the twenty-first century. The present article uses a composite of presenting case symptoms to hypothetically illustrate differential diagnoses of pain of the tongue and why there may, or may not be, links to aspects of infection of SARS-CoV-2 (COVID-19).

## Key points

- Geographic tongue is common, rarely symptomatic and not potentially malignant.
- Psychological upset can be an aggravating factor for a burning-like sensation in the mouth.
- Altered taste, in the absence of a COVID-19 infection, is usually the consequence of plaque-induced disease or longstanding oral dryness.

## Introduction

A 54-year-old man was referred to an oral medicine service by his general medical practitioner (GMP) as the patient had been experiencing a stinging-like sensation of the dorsum of the tongue for the past four months. He reported previous sore red patches that seemed to arise and diminish spontaneously for many years but the pain that was causing the patient most distress was the continuous stinging sensation across the dorsum of the tongue. The GMP had initially assumed all the symptoms were due to 'thrush' but as nystatin had not lessened the symptoms or signs, he had checked the full blood cell count, haematinics (ferritin, serum vitamin B12 and serum and red cell folate) and random glucose and found these to be normal.

The attending clinicians established that the patient had been experiencing intermittent soreness of the dorsum of the tongue over the past 30 years. The symptoms comprised intermittent episodes of mild stinging across different areas of the dorsum that accompanied the emergence of red patches that varied in site and size on the dorsum. The stinging was particularly noticeable when the patient ate tomatoes and/or pizza. However, separate to this, over the past four months, the patient had experienced a mild, burning-like sensation across the anterior dorsum of the tongue and noticed a constant metallic-like taste. This unusual taste was present most of the time, generalised throughout the mouth, had no identifiable local initiating factor, but would lessen for a short time following eating or drinking. The taste disturbance did not correlate with any possible acute COVID-19 illness. The burning-like sensation had started to reduce in the past two weeks but was arising on the anterior half of the dorsum of the tongue. The patient did not report having a dry mouth or any other abnormal sensations to the mouth.

The patient was an accountant and married with two children, aged 6 and 12 years. He did not smoke tobacco and drank about seven units of white wine per week. He had no notable features in his medical history. He had not had symptoms suggestive of acute or long COVID disease and had received the second dose of the AstraZeneca (ChAdOx1 nCoV-19) vaccine three months before the worsening of the tongue discomfort and onset of the taste disturbance. His wife and children had not had any symptoms suggestive of acute or long COVID disease. The patient seemed mildly anxious and worried. Clinical examination revealed no cervical lymphadenopathy or enlargement of the major salivary glands. Intraorally, the mouth was not dry and there were no features of long-standing oral dryness, nor evidence of plaque-induced gingival or periodontal disease. All permanent teeth were present other than the third molars (these had been extracted many years ago). The dorsum of the tongue was mildly fissured, particularly at the lateral aspects and there were some areas of circular red areas with a slight yellow-to-white border. The tongue was soft and mobile and there was no evidence of oral mucosal or gingival speckling. There was no clinically obvious local cause for the dysgeusia or burning sensation of the tongue.

The working diagnosis of the red patches and fissuring of the tongue was erythema migrans (geographic tongue), while the working diagnosis of the burning sensation of the tongue and dysgeusia was burning mouth syndrome (BMS). There was no requirement for additional investigations as the only relevant haematological/serological investigations had been found to be normal by the patient's GMP and there were no logical differential diagnoses that required to be considered. Treatment comprised providing the patient with clear information concerning both diagnoses (detailed later). There was no need for any additional intervention with regard to the BMS, while with regard the erythema migrans, the patient was advised to avoid, if possible, foods that caused discomfort to the tongue and to dab benzydamine hydrochloride onto any areas that caused distress.

The patient was reviewed three months later, at which time, he reported that the symptoms and signs of the erythema migrans were continuing but he was now endeavouring to avoid eating foods that worsened the discomfort. The burning-like sensation and metallic taste had gradually dissipated.

## Discussion

The COVID-19 pandemic has had a significant adverse impact upon all aspects of life across the globe. With regard to oral healthcare, the greatest impact has been the cessation of aerosol generating procedures, the need for social distancing within the clinical setting and the need for fallow times when operative clinical services recommenced. There are many other downstream effects upon oral healthcare that include: increased disease burden (including malignancy); limited or no immediate patient access to oral health care; loss of income and employment opportunities of staff; and a fall in morale of many oral healthcare providers. Each of these are well-detailed elsewhere^1^ but one key aspect that has received scant attention is how little acute COVID disease directly affects the mouth - other than giving rise to altered taste and smell - and how little is known about how long COVID disease will impact upon the mouth and oral health care.

Certainly, in the early phases of the COVID-19 pandemic, it was suggested that up to 60% of infected individuals had altered smell and/or taste sense. These varied between patients and could comprise complete loss of smell (anosmia) and/or taste (ageusia), partial loss (hyposmia; dysgeusia) or distortion of taste (parosmia). No one taste sense is particularly affected.^2^ The precise duration varied between individuals and could, or can be, long-standing and hence considered part of long COVID disease. The precise cause of this altered sensation is unknown but probably reflects direct viral damage to, for example, the olfactory bulb or neurons of the cleft,^3^^,^^4^ possibly having genetically determined risk factors.^5^ As yet, there remains no known therapy for loss of taste and/or smell - now termed post-infectious olfactory function - although a number of strategies to treat this have been suggested.^6^ Anecdotally, there is evidence that infection with the omicron variant of COVID-19 does not give rise to the altered taste and/or smell as frequently as that of the delta variant.

The present patient reported the onset of dysgeusia and a burning sensation. As the sensation was of a burning quality that extended across the entire dorsum, had no identifiable precipitants or relieving factors, was unrelated to the geographic tongue and accompanied by dysgeusia, it was appropriate to consider the overall diagnosis as BMS. This disorder is not uncommon, tends to arise in middle-to-late life, may be more common in women than men and can be associated with a wide range of potential aggravating factors.^7^ A number of such possible aggravating non-physical factors have emerged as a consequence of the potential effect of the COVID-19 pandemic upon individuals, their families, and society, hence it is probable that more patients than ever will develop symptoms akin to BMS.

Prior to the onset of the COVID-19 pandemic, the symptom of a persistent altered taste was considered uncommon. Most taste changes are short term, reflect local causes (such as foods, entrapment of food between teeth or in carious cavities, plaque-induced gingival disease [for example, acute necrotising ulcerative gingivitis] or acute periodontal disease) and do not give rise to an altered taste throughout the mouth. A persistent and generalised altered taste may be a feature of long-standing oral dryness (of many causes). A metallic taste can arise with some drugs, for example, metronidazole, but the present patient did not have plaque-induced oral disease, was not receiving medication and did not have features of a long-standing dry mouth.^8^

While there are many other, usually rare, causes of altered taste, it was concluded that this symptom reflected some likely distress - as reflected by the accompanying burning/stinging-like sensation of the tongue. This was based upon: 1) the patient was outwardly worried; 2) there was no local or common systemic explanation for the symptoms; 3) there was no correlation with any acute or long COVID disease; and 4) the taste sensation was metallic in quality. In BMS, the dominant symptom is one of burning that may be transiently relieved by eating and/or drinking but not improved with simple analgesics. This is often accompanied by other features; indeed, these are often the more frequent and/or dominant and may include a burning-like sensation of the tongue and other intraoral sites, a sensation of oral dryness in the absence of signs of a lack of saliva (and/or a paradoxical feeling of 'too much saliva') and/or sensation of a lump or restriction in the pharynx.^7^

There remains no one specific therapy for BMS and related symptoms and while the literature suggests that this is often a problem that may persist for many years, it is often aggravated by a variety of factors, - known or unknown to the patient. While aggravating factors (for example, financial, interpersonal, medical, many others) cannot be miraculously resolved, it is always possible to inform the patient of the diagnosis and indicate that there is no overt disease of the mouth (or indeed elsewhere) causing this symptom. Indeed, sometimes this is enough to dampen down the symptom or indeed resolve it. Where this is not possible, there are a variety of strategies that can be applied in the specialist setting to aid the patient.^7^ In the present instance, the burning sensation and metallic taste abated gradually over a three-month period. The possible stressors were not explored but in the age of the COVID-19 pandemic, there are many to consider, each possibly unique to an individual. Note that at no point has the term 'reassurance' been used. This is perhaps an overly used term in healthcare. Reassurance assumes that a patient is indeed reassured by information - a more appropriate phrase might be 'information was provided that met the patient's present self-reported needs'.

Erythema migrans (geographic tongue) is common, affects all genders and ethnicities and probably arises in childhood or early adulthood. The signs (patches of erythema, usually of the dorsum of tongue) may occasionally be brought on by certain foods (for example, chocolate or cheese) and the symptom of stinging may be worsened by hot, citrus or spicy agents. It has few associations with systemic disease. Many individuals with erythema migrans have no associated symptoms and only develop discomfort when they change their diet (for example, eat foods with tomatoes) but for those with symptoms, there is no specific therapy other than avoidance of possible precipitants and application of a local analgesic agent (for which there is no evidence-base). Certainly, this disorder is not due to fungal infection, is not potentially malignant and not related to acute or long COVID disease.

Of interest, perhaps, is that some national newspapers suggested that erythema migrans was a feature of acute COVID disease - it is not. Acute COVID disease probably makes little direct impact upon the mouth. The small number of reports of possible manifestations of acute COVID are not notably detailed, although areas of non-specific erythema and ulceration of the oral mucosa have been described. Extensive ulceration, probably due to erythema multiforme, has been reported, which is perhaps unsurprising given the wide array of drugs that patients may receive for the management of SARS-CoV-2 infection.^9^^,^^10^^,^^11^ Oral dryness, described as 'xerostomia', is likewise not unexpected, as is gingival swelling, probably due to plaque. At present, the only consistent and well-described oral feature of such infection is post-COVID-19 rhino-orbito-cerebral mucormycosis that has affected individuals in parts of India. This systemic mycotic may cause trigeminal neuropathy and 25% of affected persons have had destruction of palatal bone. It has arisen on a background of diabetes mellitus, high-dose corticosteroids, long-term hospitalisation and vaccination.^12^^,^^13^^,^^14^^,^^15^ COVID-19 vaccines very rarely give rise to any adverse effects upon the mouth.^16^^,^^17^

This case demonstrates a number of key points relevant to oral health care: 1) erythema migrans is common but can be confused sometimes with other disorders that cause pain of the tongue; 2) burning mouth syndrome can be accompanied by a gamut of oral symptoms other than just pain; 3) distress, caused or aggravated by many factors, can give rise to BMS and related disorders; and finally 4) while acute and long COVID disease have little direct effect upon oral function, other than causing dysgeusia, the very many concerns of living in the age of COVID-19 may sometimes give rise to unusual oral symptoms.
